# Impairment of cognitive functioning during Sunitinib or Sorafenib treatment in cancer patients: a cross sectional study

**DOI:** 10.1186/1471-2407-14-219

**Published:** 2014-03-24

**Authors:** Sasja F Mulder, Dirk Bertens, Ingrid ME Desar, Kris CP Vissers, Peter FA Mulders, Cornelis JA Punt, Dick-Johan van Spronsen, Johan F Langenhuijsen, Roy PC Kessels, Carla ML van Herpen

**Affiliations:** 1 Department of Medical Oncology, Radboud University Medical Centre, PO Box 9101, 6500 HB Nijmegen, The Netherlands; 2 Department of Medical Psychology, Radboud University Medical Centre, PO Box 9101, 6500 HB Nijmegen, The Netherlands; 3 Donders Institute for Brain, Cognition and Behaviour, Radboud University Nijmegen, PO Box 9104, 6500 HE Nijmegen, The Netherlands; 4 Department of Anesthesiology, Pain and Palliative Medicine, Radboud University Medical Centre, PO Box 9101, 6500 HB Nijmegen, The Netherlands; 5 Department of Urology, Radboud University Medical Centre, PO Box 9101, 6500 HB Nijmegen, The Netherlands; 6 Department of Medical Oncology, Academic Medical Centre, University of Amsterdam, PO Box 22660, 1100 DD Amsterdam, The Netherlands; 7 Department of Internal Medicine, Canisius Wilhelmina Hospital, PO Box 9015, 6500 Nijmegen, The Netherlands

**Keywords:** Cognitive function, Sunitinib, Sorafenib, VEGFR TKI, Memory and Learning, Executive functioning

## Abstract

**Background:**

Impairment of cognitive functioning has been reported in several studies in patients treated with chemotherapy. So far, no studies have been published on the effects of the vascular endothelial growth factor receptor (VEGFR) inhibitors on cognitive functioning. We investigated the objective and subjective cognitive function of patients during treatment with VEGFR tyrosine kinase inhibitors (VEGFR TKI).

**Methods:**

Three groups of participants, matched on age, sex and education, were enrolled; 1. metastatic renal cell cancer (mRCC) or GIST patients treated with sunitinib or sorafenib (VEGFR TKI patients n = 30); 2. patients with mRCC not receiving systemic treatment (patient controls n = 20); 3. healthy controls (n = 30). Sixteen neuropsychological tests examining the main cognitive domains (intelligence, memory, attention and concentration, executive functions and abstract reasoning) were administered by a neuropsychologist. Four questionnaires were used to assess subjective cognitive complaints, mood, fatigue and psychological wellbeing.

**Results:**

No significant differences in mean age, sex distribution, education level or IQ were found between the three groups. Both patient groups performed significantly worse on the cognitive domains Learning & Memory and Executive Functions (Response Generation and Problem Solving) compared to healthy controls. However only the VEGFR TKI patients showed impairments on the Executive subdomain Response Generation. Effect sizes of cognitive dysfunction in patients using VEGFR TKI were larger on the domains Learning & Memory and Executive Functions, compared to patient controls. Both patients groups performed on the domain Attention & Concentration the same as the healthy controls. Longer duration of treatment on VEGFR TKI was associated with a worse score on Working Memory tasks.

**Conclusions:**

Our data suggest that treatment with VEGFR TKI has a negative impact on cognitive functioning, specifically on Learning & Memory, and Executive Functioning. We propose that patients who are treated with VEGFR TKI are monitored and informed for possible signs or symptoms associated with cognitive impairment.

**Trial registration:**

ClinicalTrials.gov Identifier: NCT01246843.

## Background

Cognitive complaints have been reported in cancer patients treated with chemotherapy, which has been confirmed by objective neuropsychological assessment [1-3]. Several candidate mechanisms have been suggested, such as direct neurotoxic effects of chemotherapy, oxidative damage, immune dysregulation, microemboli and genetic predisposition [4]. To date no studies have been published on the effects of targeted drugs, such as the vascular endothelial growth factor receptor tyrosine kinase inhibitors (VEGFR TKI) sunitinib and sorafenib on cognitive functioning. The vascular endothelial growth factor (VEGF) plays an important role in the biology of the central nervous system. Angiogenetic factors, especially VEGF, are involved in neurogenesis, neuroprotection and the pathogenesis of stroke, Alzheimer’s disease and motor neuron disease [5]. In patients with Alzheimer’s disease the mean serum VEGF concentration is significantly lower than in healthy controls and the lower the VEGF level the higher the risk for Alzheimer’s disease [6]. Results of research with rodents indicate that VEGF expression in the hippocampus is a mediator of the effects of the environment on neurogenesis and cognition, learning and memory [7,8].

Besides VEGF, cytokines are also involved in the functioning of the central nervous system [4]. Several studies have reported a relationship between cognitive impairment and cytokine levels [4,9-12]. Higher interleukin-6 (IL-6) levels were associated with cognitive impairments on the domain Executive Functions, whereas higher IL-8 levels were associated with better Memory performance. IL-6, IL-1 receptor antagonist and tumour necrosis factor alpha (TNF-α) levels were related to ratings of fatigue [12].

Only two case reports have been published on neurobehavioral dysfunction during treatment with sunitinib, but these did not include standardized neuropsychological assessment tools [13,14]. The first paper describes three patients with preexisting cerebrovascular changes who developed severe cognitive and behavioral disorders during sunitinib treatment, which normalized within one week after discontinuation of sunitinib [13]. The second paper reports two patients who developed severe psychotic symptoms in the course of sunitinib treatment which also disappeared after cessation of the drug [14]. No studies have been performed however, examining milder forms of cognitive impairments using validated neuropsychological tests during VEGFR TKI treatment.

This prompted us to examine cognitive functioning and assess subjective cognitive complaints in patients using the VEGFR TKI sunitinib or sorafenib. Since objective cognitive dysfunction has also been reported in untreated cancer patients [12,15-17], patient controls were included. We conducted a cross sectional study with three study groups: patients with metastatic renal cell cancer (mRCC) or gastrointestinal stromal tumors (GIST) treated with the VEGFR TKI sunitinib or sorafenib, patients with mRCC without systemic treatment and healthy controls.

## Methods

### Participants and procedure

Thirty patients with mRCC or GIST treated with sunitinib or sorafenib for at least 8 weeks (VEGFR TKI patients), as well as 20 patients with mRCC, not receiving systemic treatment and previously not treated with a VEGFR TKI (patient controls), were selected to participate in this cross sectional study. Furthermore, 30 healthy controls were included as reference group from the same socioeconomic background in order to match patients and controls on four important characteristics (age, sex, estimated IQ and level of education), which in itself affect cognitive performance, and cannot be properly adjusted for statistically. Patients were recruited through their treating specialist; controls were recruited among the acquaintances of the patients and by advertisements in local papers. Eligibility criteria included: age ≥18 years, Karnofsky Performance Status (KPS) ≥70 and fluent in the Dutch language. Participants were excluded if they had been treated with systemic chemotherapy or interferon alpha (IFN-α) or IL-2 during the last 12 months, had general anesthetics in the last 3 months, were known with brain metastasis, brain injury, cognitive disorders, or psychiatric or anti-epileptic drug use. Age, sex, level of education using a 7 point scoring system (1: less than primary school, 7: university degree) [18] and estimated IQ were used for matching purposes. The study was approved by the Medical Review Ethics Committee Region Arnhem-Nijmegen and all participants gave written informed consent.

### Neuropsychological tests and self-report questionnaires

An extensive neuropsychological assessment, duration approximately 90 minutes, was administered by a trained neuropsychologist. The assessment consisted of 12 sensitive Dutch versions of widely used and well-validated tests covering the major cognitive domains, that is, Learning & Memory, Attention & Concentration, and Executive Functions. Tests in each domain were selected on the basis of cognitive theory and clinical validation studies and covered all relevant subdomains [19]. First, within the domain Learning & Memory, Working Memory was assessed by the subtests Digit Span Backwards and Letter-Number Sequencing of the WAIS-III [19]; Episodic Memory was measured using the Rey Auditory Verbal Learning Test (RAVLT) [19] and the subtest Story Recall of the Rivermead Behavioural Memory Test (RBMT) [19] and Semantic Memory was assessed by the Semantic Fluency Test (animal/profession naming) [19]. As part of the domain Attention & Concentration, Sustained Attention was assessed using the d2 Test [20], Alertness (attention span) was measured by the WAIS-III Digit Span Forward [19] and the subtest Alertness from the computerized TAP 2.1 [21]. In the domain Executive Functions the Controlled Oral Word Association Test (COWAT) [19] was used to measure Response Generation. Response Inhibition was tapped by the Stroop Color-Word test (interference score) [19], Mental Flexibility by the subtest Flexibility of the TAP 2.1 [20] and Problem Solving by the Brixton Spatial Anticipation Test [19] and Raven’s Advanced Progressive Matrices (Set I) [19]. To estimate the level of premorbid intelligence the Dutch version of the National Adult Reading Test (NART) [19] was administered. Moreover, self-report questionnaires were administered to assess psychological well-being (Symptom Checklist–Revised; SCL-90-R) [22], everyday cognitive failures (Cognitive Failures Questionnaire; CFQ) [23], mood (Beck Depression Inventory–Second Edition; BDI-II) [24] and fatigue (Checklist Individual Strength; CIS20r) [25].

### Biomarkers

In the patient groups, blood samples were obtained on the day of the neuropsychological assessment. Blood samples were analyzed for a full blood count, liver and renal function, and levels of testosterone, sex hormone binding globuline (SHBG), estradiol, albumin, vitamin B12, thyroid function, glucose C-reactive protein (CRP), erythrocyte sedimentation rate (ESR) and lactate dehydrogenase (LDH). Free testosterone was calculated from the testosterone and SHBG values. [26] Plasma VEGF–and serum cytokine levels were measured. Levels of VEGF were measured by a specific ELISA as previous described [27,28]. We used the Th1/Th2 11plex kit (eBioscience) according to the manufacturer’s protocol to measure cytokines levels. The minimum detectable concentrations were estimated to be 4.2 pg/ml for IL-1β, 16.4 pg/ml for IL-2, 20.8 pg/ml IL-4, 1.6 pg/ml IL-5, 1.2 pg/ml IL-6, 0.5 pg/ml IL-8, 1.9 pg/ml IL-10, 1.5 pg/ml IL-12 (p70), 3.2 pg/ml TNF-α, 2.4 pg/ml TNF-β and 1.6 pg/ml interferon gamma (IFN-γ). Results were expressed as percentage of detectable values and as median values in both patient groups.

### Statistical analyses

All neuropsychological tests were scored according to their manuals. For data reduction purposes and to enhance the comparability of cognitive (sub)domains, standardized z-scores were computed using the raw test results. All analyses were performed on these (sub)domain scores. The performances on the individual (sub)tests are presented for descriptive purposes only. For the self-report questionnaires, total and subscale scores were calculated using their manuals. Overall between-group analyses were performed using multivariate analysis of variance (general linear model) with Fisher’s post-hoc *t*-tests or nonparametric tests for nominal or ordinal variables (sex distribution and education level). The biomarkers were assessed for (log-)normality and *t*-tests were used to compare patient groups when applicable. Pearson’s correlation coefficients (*r*) were computed to examine relationships between cognitive performance and self-report measures on one hand and the biomarkers on the other. Analyses were conducted using SPSS 18.0 for Windows (SPSS, Chicago, IL).

## Results

### Participants

Between August 2009 and May 2011 a total of 80 patients and controls were enrolled. Within the VEGFR TKI group, 26 patients had a diagnosis of mRCC and 4 of GIST. Three patients in the VEGFR TKI group and 4 in the patient controls had been treated in the past (> 1 year before) with a combination of IFN-α, IL-2 and 5FU. One patient in the VEGFR TKI group and 2 patients in the patient control group had previously been treated with IFN-α monotherapy. During the study 23 patients were treated with sunitinib and 7 with sorafenib. The median duration of treatment with VEGFR TKI at the time of the neuropsychological assessment was 20 months (range 2-55). Most patients on sunitinib were on a continuous schedule (n = 14), while the others were treated on a 4 weeks on and 2 weeks off schedule. The dose ranged from 25 mg continuously to 50 mg 4 weeks on and 2 weeks off. Sorafenib dosing was continuously with a total daily dose of 800 mg in most patients.

### Neuropsychological tests

**Figure 1:**
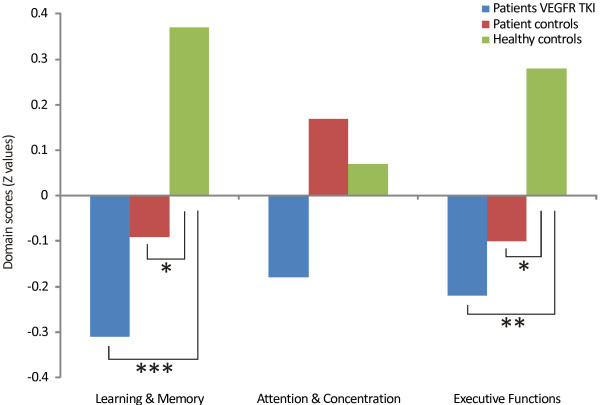
**Cognitive domain scores.** The cognitive domain scores (standardized Z values) for the patients using VEGFR TKI, the patient controls and the healthy controls. Post-hoc *t*-tests, patient groups compared to healthy controls: *P < 0.05, **P < 0.01, ***P < 0.001.

All participants were able to complete all neuropsychological tests and self-report questionnaires. Participants characteristics (age, sex distribution, estimated IQ and education level) were equally distributed among the 3 groups (Table 1), indicating that the groups were well-matched. Significant differences between the groups were found on the domains Learning & Memory (F(2,77) = 8.2, *P* = .001) and Executive Functions (F(2,77) = 4.5, *P* = .014). No significant differences were demonstrated for the domain Attention & Concentration (F(2,77) = 1.7, *P* = .20). Post-hoc comparisons showed that, compared to the healthy controls, the VEGFR TKI patients performed worse on the domain Learning & Memory (*P* = .0001) and Executive Functions (*P* = .005) (Table 2). The patient controls also performed worse than healthy controls on Learning & Memory (*P* = .019) and Executive Functions (*P* = .049). No significant differences were found between the VEGFR TKI and the patient controls on the domains Learning & Memory (*P* = .24) and Executive Functions (*P =* .55). Figure 1 shows that the magnitude of the effects were largest in the VEGFR TKI patients.

**Table 1:** Characteristics of the study groups

	**Patients VEGFR TKI (n = 30)**		**Patient controls (n = 20)**		**Healthy controls (n = 30)**		***P***
Characteristics							
Age, years							
Mean	60		62		58		0.456^e^
Range	38-81		30-75		45-73		
Sex (%)							
Male	27	(90)	15	75	26	(87)	0.329^f^
Female	3	(10)	5	25	4	(13)	
Median education level (SD)^d^	4	(1.21)	5	(1.12)	5	(0.80)	0.546^g^
Estimation IQ (SD)	102.30	(10.37)	106.20	(10.55)	106.37	(8.09)	
Median free testosterone^c^ pmol/L (IQR 25-75)	165.00	(129,5-238.00)	237.50	(200.75-299.25)	-	-	0.005^a^
Median estradiol pmol/l^†^ (IQR 25-75)	48.00	(42-55)	78.50	(78.5-112.5)	-	-	0.000 ^a^
Median albumin g/l (IQR 25-75)	39.00	(34.50-40.25)	37.50	(35.25-40.00)	-	-	0.599^a^
Median CRP mg/l (IQR 25-75)	10.00	(4.00-21.75)	5.00	(4.00-38.25)	-	-	0.849^a, b^
Median absolute neutrophil count 10^9^/l (IQR 25-75)	2.95	(2.08-3.66)	3,89	(3.43-5.64)	-	-	0.009^a^
Median ESR mm/hour (IQR 25-75)	20.00	(8.75-34.50)	19.00	(7.00-31.00)	-	-	0.767^a, b^
Median glucose mmol/l (IQR 25-75)	5.6	(4.9-5.98)	5.8	(5.35-7.55)	-	-	0.010^a^
Median TSH mE/l (IQR 25-75)	2.29	(1.49-3.63)	1.12	(0.78-2.04)	-	-	0.003^a, b^
Median LDH U/l (IQR 25-75)	463.50	(402.25-526.50)	346.50	(314.50-458.00)	-	-	0.008^a, b^
Median VEGF ng/ml (IQR 25-75)	1.62	(1.09-2.18)	1.48	(1.21-2.00)	-	-	0.221^a^

^a^T-test between the two patients groups; ^b^calculated with the logaritmic values.

^c^calculated free testosterone only in men, reference value for men: 120-630 pmol/l, ^†^estradiol level only in men, reference value 75-220 pmol/l. *Abbreviations:SD* Standard deviation, *IQR* inter quartile range 25^e^ and 75^e^ percentile, *ESR* erythrocyte sedimentation rate, *CRP* C-reactive protein, *LDH* lactate dehydrogenase, *VEGF* vascular endothelial growth factor.

^d^Education levels as assessed using 7 categories in accordance with the Dutch educational system (1 = less than primary school; 7 = academic degree); ^e^ANOVA; ^f^Chi-square test; ^g^Kruskal-Wallis Test.

**Table 2:** Cognitive subdomain scores and raw neuropsychological test scores

	**Patients VEGFR TKI**		**Patient controls**		**Healthy controls**	
	***Mean***	***SD***	***Mean***	***SD***	***Mean***	***SD***
**Learning & memory**						
*Working memory*	-0.26	0.93	0.11	0.95	0.19	0.79
Digit span backwards WAIS-III	5.67	1.77	6.40	1.98	6.37	1.75
Letter-number sequencing WAIS-III	9.47	2.27	10.15	2.08	10.50	1.59
*Episodic memory*	-0.31***	0.89	-0.11*	0.74	0.38	0.56
RAVLT total score	33.70	8.01	37.55	8.25	39.77	5.76
RAVLT delayed recall	5.87	2.57	7.00	2.32	7.37	2.50
RAVLT delayed recognition	26.33	3.02	27.70	1.59	28.03	1.45
RBMT story (Immediate Recall)	9.62	2.75	9.42	3.14	11.75	2.51
RBMT story (Delayed Recall)	8.43	3.51	7.85	3.30	10.38	2.56
*Semantic memory*	-0.36***	0.97	-0.26**	0.89	0.53	0.88
Semantic fluency (Animal)	22.47	5.49	22.95	4.39	26.73	5.22
Semantic fluency (Profession)	16.30	4.65	16.80	5.19	20.70	4.21
**Attention & concentration**						
*Alertness*	-0.17	0.88	0.17	0.68	0.06	0.59
Digit span forwards WAIS-III	8.23	2.05	9.20	1.94	8.47	1.28
TAP 2.1 alertness^a^	256.28	51.96	248.40	35.52	240.75	27.45
*Sustained attention*	-0.22	0.93	0.18	1.16	0.10	0.94
d2 test	138.63	30.93	151.60	38.33	149.03	31.25
**Executive functions**						
*Response generation*	-0.26*	0.91	-0.13	1.23	0.35	0.83
Letter fluency (COWAT)	32.97	9.28	34.25	12.58	39.17	8.46
*Response inhibition*	-0.04	1.05	0.01	1.05	0.04	0.95
Stroop color word test (Interference)^a^	0.70	0.30	0.73	0.47	0.65	0.21
*Mental flexibility*	-0.29	0.98	0.11	1.13	0.22	0.88
TAP flexibility (Alternation)^a^	900.37	353.23	824.40	405.37	732.03	177.52
*Problem solving*	-0.19*	0.88	-0.18*	0.85	0.31	0.74
Raven APM (Set I)	8.00	2.23	8.10	2.02	9.50	1.93
Brixton spatial anticipation Test	36.97	6.06	36.90	6.31	38.83	5.43

Cognitive subdomain scores (standardized Z-scores, mean + SD) and raw neuropsychological test scores (mean + SD) for the patients using VEGFR TKI, the patient controls and the healthy controls. Higher scores indicate a better performance except where noted. Post-hoc *t*-tests, patient groups compared to healthy controls, for the cognitive subdomain scores: *P < 0.05, **P < 0.01, ***P < 0.001. ^a^Higher scores reflect a worse performance. *Abbreviations:RAVLT* Rey Auditory Verbal Learning Test, *RBMT* Rivermead Behavioural Memory Test, *WAIS-III* Wechsler Adult Intelligence Scale–Third Edition, *TAP* Test for Attentional Processing, *Raven APM* Raven Advanced Progressive Matrices.

Subsequently, analyses were performed between the three groups, on the cognitive subdomains for the significant domains Learning & Memory and Executive Functions (Table 2). With respect to the domain Learning & Memory, between-group differences were observed on Episodic Memory (F(2,77) = 6.7, *P* = .002) and Semantic Memory (F(2,77) = 8.1, *P* = .001), no differences were found on Working Memory (F(2,77) = 2.1, *P* = .13). Post-hoc comparisons showed that both the VEGFR TKI patients and the patient controls performed worse than healthy controls on Episodic Memory (*P* = .001 and *P* = .03) and Semantic Memory (*P* < .0001 and *P* = .004). Within the domain Executive Functions, between-group differences were found on Problem Solving (F(2,77) = 3.5, *P* = .037) and Response Generation (F(2,77) = 3.2, *P* = .047), no differences were found on Inhibition (F(2,77) = .04, *P* = .96 and Mental Flexibility (F(2,77) = 2.2, *P* = .12). Post-hoc comparisons showed that the VEGFR TKI patients performed worse than healthy controls on both Problem Solving (*P* = .02) and Response Generation (*P* = .02). The patient controls performed worse on Problem Solving (*P* = .04) compared to the healthy controls. In the VEGR TKI group, longer treatment on VEGFR TKI was associated with a worse score on Working Memory tasks (*r* = -.461 *P* = .012).

### Self-report questionnaires

With respect to the self-report questionnaires, significant between-group differences were found on psychological well-being as measured with the SCL-90-R (Table 3) (F(2,77) = 7.5, *P* = .001), mood scores as assessed with the BDI-II (Table 3) (F(2,77) = 12.9, *P* = .000) and fatigue measured with the CIS20r (Table 3) (F(2,77) = 7.2, *P* = .001). No between-group differences were found on experienced cognitive failures in daily functioning assessed with the CFQ (Table 3) (F(2,77) = .7, *P* = .50). Post-hoc comparisons showed that on psychological well-being the VEGFR TKI patients reported more feelings of anxiety (*P* = .005), depressive symptoms (*P* < .0001), somatic symptoms (*P* < .0001) and subjective cognitive complaints (*P* = .002), as well as an overall heightened level of distress (*P* = .001) compared to the healthy controls. The patient controls reported more symptoms than healthy controls on the subscales anxiety (*P* = .034), depressive symptoms (*P* = .003), somatic symptoms (*P* = .029), subjective cognitive complaints (*P* = .005), and the total distress scale (*P* = .004).

**Table 3:** Self-reported psychological well-being, subjective cognitive complaints, depressive symptoms and level of fatigue

	**Patients VEGFR TKI**		**Patient controls**		**Healthy controls**	
	***Mean***	***SD***	***Mean***	***SD***	***Mean***	***SD***
*SCL-90-R*						
Anxiety	13.07**	3.10	12.75*	3.06	11.10	1.71
Depression	23.87***	6.31	22.55**	5.32	18.20	2.68
Sleep disturbance	4.93	2.57	5.00	2.03	4.10	1.40
Agoraphobia	7.90	2.01	8.10	2.20	7.40	0.77
Somatization	19.23***	5.73	17.40*	6.07	14.17	3.18
Cognitive-performance difficulty	14.77**	4.68	14.75**	4.82	11.43	2.13
Interpersonal sensitivity and paranoid ideation	20.90	3.63	22.50	5.69	20.87	3.15
Anger-hostility	6.90	1.27	6.90	1.12	6.43	0.86
Total score	123.10***	24.13	121.80**	26.81	103.67	11.88
*CFQ*						
Total score	93.37	13.20	89.55	10.13	91.80	9.64
*BDI-II*						
Total score	9.87***	5.76	8.25**	5.78	3.60	2.98
*CIS20r*						
Total score	67.67***	26.75	61.05*	21.11	46.60	16.24

Self-reported psychological well-being, subjective cognitive complaints, depressive symptoms and level of fatigue (mean + SD) for the patients using VEGFR TKI, the patient controls and the healthy controls. Post-hoc *t-*tests, patient groups compared to healthy controls: *P < 0.05, **P < 0.01, ***P < 0.001. *Abbreviations:SCL-90-R* Symptom Checklist-Revised, *CFQ* Cognitive Failures Questionnaire, *BDI-II* Beck Depression Inventory-Second Edition, *CIS-20-R* Checklist Individual Strength-Revised.

Mood scores as assessed with the BDI-II (Table 3) were higher in the VEGFR TKI group (*P* < .0001) and the patient controls (*P* = .002) compared to healthy participants. Seven (23%) VEGFR TKI patients, 2 (10%) patient controls and none of the healthy volunteers had scores above the cut-off value of 16 indicative for a depressive disorder that has been validated on advanced cancer patients [29]. Moreover, the VEGFR TKI patients and the patient controls experienced more fatigue than healthy controls on the CIS20r (*P* = .000 and *P* = .025 respectively) (Table 3). No significant differences between the two patient groups were found on any of the self-report questionnaires or subscales (Table 3). In the VEGR TKI group, longer treatment on VEGFR TKI was associated with less complaints of fatigue (CIS20r total score *r* = -.404, *P* = .030).

### Biomarkers

Between the two patients groups no significant differences were found in hemoglobin level, leucocytes and platelet counts, liver and renal function, electrolytes, HbA1c, vitamin B12 (data not shown), albumin CRP, ESR and VEGF levels (Table 1). In the VEGFR TKI patients group the calculated free testosterone- and estradiol values, absolute neutrophil counts and glucose levels were significantly lower, and the Thyrotropin (TSH) and LDH levels were higher compared to the patient controls (Table 1). No consistent correlations were found between the results of hematology- and chemistry-blood tests and the neuropsychological tests or the self-report questionnaires (data not shown). Only in the VEGFR TKI patients were higher ESR levels associated with worse scores on the main cognitive domains Learning & Memory, Attention & Concentration and Executive Functions (Table 4). CRP levels (Table 4) and higher neutrophils (data not shown) in this group were also negatively correlated with the domain score Learning & Memory. In the VEGFR TKI patients higher ESR, CRP and LDH levels were associated with higher scores on the BDI-II, indicating more depressive symptoms. No correlations were found between the free testosterone- or estradiol levels and the results on the neuropsychological tests or the self-report questionnaires (data not shown).

**Table 4:** Correlations of the biomarkers and the neuropsychological tests and self-reported questionnaires

	**Patients VEGFR TKI**		**Patient controls**		**Patients VEGFR TKI**		**Patient controls**		**Patients VEGFR TKI**		**Patient controls**		**Patients VEGFR TKI**		**Patient controls**	
	**CRP**				**ESR**				**LDH**				**VEGF**			
	***r***	***P***	***r***	***P***	***r***	***P***	***r***	***P***	***r***	***P***	***r***	***P***	***r***	***P***	***r***	***P***
***Neuropsychological tests***																
**Learning & memory**	-0.427	0.019			-0.410	0.030										
* Working memory*																
* Episodic memory*	-0.432	0.017														
* Semantic memory*																
**Attention & concentration**					-0.440	0.019										
* Alertness*																
* Sustained attention*																
**Executive functions**					-0.376	0.049										
* Response generation*																
* Response inhibition*																
* Mental flexibility*																
* Problem solving*																
***Self-reported questionnaires***																
**SLC90-R total score**															-0.626	0.004
Anxiety															-0.526	0.021
Depression															-0.518	0.023
Sleep disturbance																
Agoraphobia																
Somatization															-0.476	0.039
Cognitive-performance difficulty															-0.540	0.017
Interpersonal sensitivity and paranoid ideation															-0.638	0.003
Anger-hostility															-0.729	0.000
**CFQ total score**															-0.754	0.000
**BDI-II total score**	0.379	0.039			0.378	0.047			0.464	0.010					-0.622	0.004
**CIS20r total score**																
CIS fatigue																
CIS motivation																
CIS concentration															-0.466	0.045
CIS activation																

Correlations between the biomarkers and the Neuropsychological Tests and Self-report Questionnaires in the VEGFR TKI patients and the patient controls. Only the significant correlations are listed in the table. *r* = Pearson correlation coefficient.

In both patient groups, the VEGF levels were not associated with the results on the cognitive domain scores or fatigue (CIS20r) (Table 4). Only in the patient control group higher VEGF levels were associated with less complaints on mood (BDI-II), psychological well-being (SLC-90-R) and cognitive failure in daily functioning (CFQ) (Table 4).

We were able to analyze serum cytokine levels in 29 VEGFR TKI patients and 18 patient controls. In both groups no detectable levels of IL-5 and IL-6 were found in any of the patients, and IL-2 and TNF- α levels were only sporadically detected (data not shown). The IL-8 level was detectable in 80% (n = 23) of the VEGFR TKI group and in 67% (n = 12) of the patient controls and no difference was found in IL-8 levels between the patient groups. We found no correlations between the serum IL-8 level and the scores on the neuropsychological tests or the self-report questionnaires.

No correlations were found between the duration of treatment with VEGFR TKI and biomarker concentrations (data not shown) or the results of the neuropsychological tests and the self-report questionnaires, except for the results on the subdomain Working Memory and the CIS20r.

## Discussion

This study is the first to examine cognitive functioning and subjective cognitive complaints in cancer patients during treatment with the VEGFR TKI sunitinib or sorafenib. We found that these patients performed worse on the cognitive domains Learning & Memory (Episodic-and Semantic Memory) and Executive Functions (Response Generation and Problem Solving) compared to healthy controls. Furthermore, a longer duration of VEGFR TKI treatment was associated with worse functioning on Working Memory tasks. Patient controls also showed impairments on the neuropsychological tests concerning Learning & Memory (Episodic- and Semantic Memory). However, in contrast with the VEGFR TKI patients, they showed impairment only on the subdomain Problem Solving but not on Response Generation. Our data suggest that effect sizes of cognitive dysfunction in patients using VEGFR TKI are larger on the domains Memory & Learning and Executive Functions, compared to patient controls. Although we found no significant differences in the results of the neuropsychological tests between the VEGFR TKI patients and the patient controls, possibly due to the smaller group size of the patient control group.

Since both patient groups performed on the domain Attention & Concentration the same as the healthy controls, the observed deficits in the other domains are not due to worse attention and concentration.

On self-reported psychological well-being, subjective cognitive complaints, depressive symptoms and fatigue, both patient groups reported significantly more complaints compared to the healthy controls. Although the VEGFR TKI patients showed more cognitive impairments on the domain Executive Functions, both patient groups reported equal levels of psychological and somatic complaints on the self-report questionnaires. Moreover, the non-significant, yet slightly higher scores on depressive symptoms and fatigue of the VEGFR TKI patients do not explain the lower scores on the memory and executive functioning tests. That is, no differences were found between the patient groups and the healthy control group on attention and concentration tasks, which are typically susceptible for mood disturbances and fatigue [19].

We did not observe any consistent correlations between self-reported cognitive complaints and neuropsychological measures neither in patients, nor in healthy controls (data not shown), as is observed in patients treated with chemotherapy [9,30,31].

We chose to perform a cross sectional study design, which is frequently used in neuropsychology, as this could give an indication if cognitive functioning was indeed decreased. Our study design included two relevant and well matched control groups and the results were not confounded by practice effects through repeated testing. Furthermore, the in vitro rodent data demonstrated that VEGF plays a role in cognitive functioning [7,8]. The complaints of VEGF TKI treated patients about cognitive functioning and the result of this study support the necessity for a longitudinal study on cognitive functioning in these patients.

We included both patients on sunitinib and sorafenib. As both sunitinib and sorafenib inhibit the VEGFR2 [32,33], and we presumed that the cognitive functioning would be influenced by blocking this pathway, there was no reason to exclude one of both angiogenesis inhibitors. We did not perform a MRI of the brain before inclusion but Included patients did not have symptomatic brain metastases. Therefore, we may have missed asymptomatically brain metastases, although there is no reason to expect that this was different between the two patient groups.

In our study we explored factors possibly influencing cognitive functioning in cancer patients and specifically in patients during VEGFR TKI treatment. We demonstrated that male patients on treatment with sunitinib or sorafenib had lower free testosterone levels compared to patient controls, possibly due to the treatment. However we observed no relation between these sex hormones and cognitive functioning. Previous studies correlating cognitive functioning with testosterone levels in hypogonadal men and studies on androgen-ablation therapy have produced inconsistent results [34,35].

In contrast to others [12], we found no correlation between serum IL-8 concentrations and objective or subjective cognitive functioning. In mRCC patients elevated levels of serum IL-6 and IL-8 [36-38], neutrophil counts and LDH [39] have been identified as markers of a systemic inflammatory response and predictors of worse prognosis. In the VEGFR TKI patients we found that higher levels of ESR, CRP and neutrophils were associated with worse objective cognitive functioning, and higher levels of ESR, CRP en LDH with depressive symptoms. Especially the ESR level seems relevant as it showed correlations with all cognitive domains. Our data suggest that markers of systemic inflammatory response, probably as a symptom of tumor progression, are correlated with worse cognitive performance and more depressive feelings in patients treated with VEGFR TKI. This is consistent with the work of others who found that higher CRP levels were associated with depression and worse cognitive functioning [40,41].

Recently a review was published addressing the role of VEGF in the brain and the role of VEGF inhibitors on cognitive impairment. Ng et a. concluded that VEGF plays an important role in the Central Nervous System such as neurogenesis and neuroprotection, and that studies suggest that VEGF may affect cognitive functioning through its effects on neurogenesis, cerebral blood flow and modulation of long-term potentiation [42]. We demonstrated no differences in plasma VEGF concentration between the two patient groups, and no influence of VEGF levels on cognitive functioning was observed. However, in the VEGFR TKI group the intracellular effect of VEGF is prevented by receptor blockade, and therefore VEGF plasma concentrations are not reflecting the intracellular concentrations and effects of VEGF in this group. A possible explanation for the difference in cognitive functioning between the two patient groups is that, as a result of blocking the cerebral VEGF receptor through the VEGFR TKI, the capacity of neuronal repair and neurogenesis and learning is decreased. Furthermore, in the patient controls we found a strong negative correlation between subjective complaints and VEGF concentration, suggesting that VEGF indeed is important for psychological well-being.

## Conclusions

In summary, our data suggest that treatment with VEGFR TKI has a negative impact on cognitive functioning, and that subjective complaints can be corroborated by objective neuropsychological testing. However this should be confirmed in a longitudinal study. Our results also warrant further studies on the underlying mechanism of the impairment of cognitive functioning during VEGF TKI therapy for example with functional imaging such as dynamic MRI imaging.

We propose that patients who are treated with VEGFR TKI are monitored and informed for possible signs or symptoms associated with cognitive impairment.

## Abbreviations

VEGFR TKI: Vascular endothelial growth factor receptor tyrosine kinase inhibitors; mRCC: Metastatic renal cell cancer; GIST: Gastrointestinal stromal tumors; VEGF: Vascular endothelial growth factor; IL-6: Interleukin-6; TNF-α: Tumour necrosis factor alpha; KPS: Karnofsky Performance Status; IFN-α: Interferon alpha; RAVLT: Rey Auditory Verbal Learning Test; RBMT: Rivermead Behavioural Memory Test; COWAT: Controlled Oral Word Association Test; NART: National Adult Reading Test; CFQ: Cognitive Failures Questionnaire; BDI-II: Beck Depression Inventory–Second Edition; CIS20r: Checklist Individual Strength; SHBG: Sex hormone binding globuline; CRP: C-reactive protein; ESR: Erythrocyte sedimentation rate; LDH: Lactate dehydrogenase; IFN-γ: Interferon gamma.

## Competing interests

The authors declare that they have no competing interests.

## Authors’ contributions

SM, DB, CH, RK, KV and CP conceived the study and participated in its design. All authors were involved in data acquisition. SM, DB, RK and CH were involved in statistical analysis and SM, DB, CM, CH, RK KV and CP in data interpretation. All authors helped to draft and edit the manuscript and all authors read and approved the final manuscript.

## Pre-publication history

The pre-publication history for this paper can be accessed here:

http://www.biomedcentral.com/1471-2407/14/219/prepub
